# Comparison of *EGFR* and *K-RAS* gene status between primary tumours and corresponding metastases in NSCLC

**DOI:** 10.1038/sj.bjc.6604629

**Published:** 2008-08-26

**Authors:** A Kalikaki, A Koutsopoulos, M Trypaki, J Souglakos, E Stathopoulos, V Georgoulias, D Mavroudis, A Voutsina

**Affiliations:** 1 Laboratory of Tumor Cell Biology, School of Medicine, University of Crete, Heraklion, Crete, Greece; 2 Department of Pathology, University General Hospital of Heraklion, Heraklion, Crete, Greece; 3 Department of Medical Oncology, University General Hospital of Heraklion, Heraklion, Crete, Greece

**Keywords:** *EGFR*, *K-RAS*, mutations, primary tumour, metastasis, NSCLC

## Abstract

In non-small-cell lung cancer (NSCLC), epidermal growth factor receptor (*EGFR*) and *K-RAS* mutations of the primary tumour are associated with responsiveness and resistance to tyrosine kinase inhibitors (TKIs), respectively. However, the *EGFR* and *K-RAS* mutation status in metastases is not well studied. We compared the mutation status of these genes between the primary tumours and the corresponding metastases of 25 patients. Epidermal growth factor receptor and *K-RAS* mutation status was different between primary tumours and corresponding metastases in 7 (28%) and 6 (24%) of the 25 patients, respectively. Among the 25 primary tumours, three ‘hotspot’ and two non-classical *EGFR* mutations were found; none of the corresponding metastases had the same mutation pattern. Among the five (20%) *K-RAS* mutations detected in the primary tumours, two were maintained in the corresponding metastasis. Epidermal growth factor receptor and *K-RAS* mutations were detected in the metastatic tumours of three (12%) and five (20%) patients, respectively. The expressions of EGFR and phosphorylated EGFR showed 10 and 50% discordance, in that order. We conclude that there is substantial discordance in *EGFR* and *K-RAS* mutational status between the primary tumours and corresponding metastases in patients with NSCLC and this might have therapeutic implications when treatment with TKIs is considered.

Lung cancer is the most frequent solid tumour and represents the leading cause of cancer death throughout the developed world. Almost 70% of patients with non-small-cell lung carcinoma (NSCLC) present with locally advanced or metastatic disease at the time of diagnosis. Non-small-cell lung carcinoma is characterised by the accumulation of multiple genetic alterations (Marsit *et al*, 2004; Yokota and Kohno, 2004; Garnis *et al*, 2006). Mutations within the tyrosine kinase domain of epidermal growth factor receptor (*EGFR*) account for increased sensitivity to tyrosine kinase inhibitors (TKIs; gefitinib and erlotinib) and they are associated with prolonged overall survival (Lynch *et al*, 2004; Paez *et al*, 2004; Perez-Soler *et al*, 2004; Chou *et al*, 2005; Taron *et al*, 2005; Hirsch, 2006). However, the point mutation T790M and an insertion mutation in exon 20 were associated with resistance to TKIs (Gazdar and Minna, 2005). Furthermore, recent studies have shown that the expression of EGFR as assessed by gene copy number, mRNA and protein levels could be used to predict responsiveness to therapy with TKIs (Hirsch *et al*, 2005; Taron *et al*, 2005; Dacic *et al*, 2006; Dziadziuszko *et al*, 2006; Endo *et al*, 2006). In addition, several clinicopathological characteristics, such as adenocarcinoma histology, non-smoking history, female gender and Asian origin, are also associated with a higher probability of response to TKIs, whereas the presence of *K-RAS* mutations seems to be correlated with primary resistance to these agents (Tokumo *et al*, 2005; Tsao *et al*, 2005; van Zandwijk *et al*, 2007). Thus, an emerging issue concerning EGFR-targeted therapy is to identify the best method for selecting patients who are more likely to benefit from EGFR inhibition.

Advanced NSCLC metastasises systemically to diverse sites, such as the brain, bone, adrenal glands and liver. The classical model for the metastatic process suggests that most cells of a given primary tumour have low metastatic potential and only a few cells acquire enough somatic mutations to become metastatic (Bernards and Weinberg, 2002). An alternative model proposes that the metastatic potential is encoded in the mass of a given primary tumour that has progressed to a pre-metastatic state, after which metastases may randomly occur without any further gene expression changes (van ‘t Veer *et al*, 2002; Hynes, 2003; Van't Veer and Weigelt, 2003). Taking into account these two models, a critical issue for the treatment of metastatic NSCLC is the question of genetic variability and differences between the primary tumour and the corresponding metastases. In the majority of studies, *EGFR* and *K-RAS* status was determined on the primary tumours and there are very few data concerning those of corresponding metastases (Italiano *et al*, 2006; Matsumoto *et al*, 2006). Therefore, it is unclear whether the same *EGFR* and *K-RAS* mutations are also present in the metastatic lesions or whether clones with different mutations are responsible for the generation of metastases.

In this study, the mutation status of *EGFR* and *K-RAS* as well as the EGFR and p-EGFR expressions on the primary tumours and the corresponding metastatic lesions were evaluated in 25 patients with advanced NSCLC. The objective of this study was to investigate the prevalence of *EGFR* and *K-RAS* mutations in metastases and to examine whether these mutations and the EGFR expression patterns are discordant between the primary tumours and the corresponding metastases. Secondary objectives were to explore whether the EGFR expression pattern correlated to *EGFR* and/or *K-RAS* mutations in both the primary tumours and corresponding metastases.

## Patients and methods

### Patients

Patients, aged >18 years old, with histologically confirmed NSCLC who underwent biopsy or surgical excision of the primary tumour and the corresponding metastases were included in this retrospective analysis. Histological type was determined according to the World Health Organization criteria, and the stage of the disease corresponds to that of the time of primary diagnosis. Smoking history was obtained during the patient's first evaluation. All patients gave their informed consent for using their tumour sample for molecular and pathologic analysis. The study has been approved by the Ethics and Scientific Committees of our institution.

### DNA extraction and mutation analysis

All tumour samples were formalin-fixed paraffin-embedded tissues. Sections of a paraffin block corresponding to one representative area of the tumour were stained with haematoxylin/eosin, and the presence of tumour tissue was verified by an experienced pathologist. Subsequently, tissue samples from at least three serial sections were microdissected (piezo power Eppendorf Microdissector; Eppendorf, Germany) to ensure that specimens contained at least 80% tumour cells; sections of 5 *μ*m thickness were also collected from adjacent normal tissue when available, extracted with xylene and ethanol to remove paraffin and placed in 1% SDS/proteinase K (10 mg ml^−1^) at 56°C overnight. DNA was extracted using the MasterPure Complete DNA/RNA Purification kit (Epicentre Biotechnologies, Madison, WI, USA) according to the manufacturer's instructions. Exons 18, 19, 20 and 21 of the EGFR and exon 1 of K-RAS were sequentially amplified by two rounds of polymerase chain reaction (PCR) and subjected to direct sequencing. The PCR primers for *EGFR* amplification were as follows: 155273L23: 5′-TCCCAAACACTCAGTGAAACAAA-3′; 155348L22: 5′-TGGTCTCACAGGACCACTGATT-3′; 154838U22: 5′-TCAGAGCCTGTGTTTCTACCAA-3′; 154899U20: 5′-TCCAAATGAGCTGGCAAGTG-3′; 55634U24: 5′-AAATAATCAGTGTGATTCGTGGAG-3′; 156027L20: 5′-TGTGGAGATGAGCAGGGTCT-3′; 156107L22: 5′-GAGGCCAGTGCTGTCTCTAAGG-3′; 155750U20: 5′-GTGCATCGCTGGTAACATCC-3′; 173160L22: 5′-CAGCTCTGGCTCACACTACCAG-3′; 173076L19: 5′-CATCCTCCCCTGCATGTGT-3′; 172656U22: 5′-GCAGCGGGTTACATCTTCTTTC-3′; and 172747U19: 5′-GCT CAGAGCCTGGCATGAA-3′. The PCR primers for *K-RAS* amplification were as follows: RASU1: 5′-AGGCCTGCTGAAAATGACTGAATA-3′; RASL1: 5′-CTGTATCAAAGAATGGTCCTGCAC-3′; RASU2: 5′-AAAATGACTGAATATAAACTTGTGG-3′; RASL2: 5′-CTCTATTGTTGGATCATATTCGTC-3′. The first PCR was carried out in a total volume of 10 *μ*l contained 1/10 of the extracted genomic DNA using 1 U of Platinum Taq DNA polymerase (Invitrogen Corporation, Carlsbad, CA, USA). The initial denaturing step was at 94°C for 15 min, followed by 35 cycles of denaturing step at 94°C for 20 s, annealing step at 60°C for 30 s and extension step at 72°C for 1 min, ending with a final extension step at 72°C for 7 min. Nested PCR was carried out in a total volume of 20 *μ*l and the conditions were identical to the first PCR. The PCR products are directly sequenced by dye terminator sequencing (ABI BigDye Terminator kit, v3.1, Applied Biosystems, Foster City, CA, USA), purified by ethanol precipitation and separated by capillary electrophoresis on an ABI 3100 Avant genetic analyzer (Applied Biosystems). Sequence analysis was carried out by Seqscape software (Applied Biosystems) and manually by two reviewers (AK and AV). All sequence variations were confirmed by sequencing in both directions and by an independent PCR amplification when sufficient material was available.

The sensitivity of our methodology was evaluated by determining the minimum frequency of *EGFR* and *K-RAS* mutations required for detection in our system. This was accomplished by performing mixing experiments using cell lines with and without *EGFR* (H2073-wt-*EGFR* and HCC827-Del19-*EGFR*) or *K-RAS* (H2073-wt-*K-RAS* and A549-G12D-*K-RAS*) mutations. These experiments demonstrated that the Del19 and *K-RAS* mutations could be detected when present in 10 and 20% of the cells in the sample, respectively (data not shown).

### Immunohistochemistry for EGFR

The paraffin-embedded tissues were cut at 4 *μ*m thickness and were deposited on SuperFrost/Plus Slides (O.Kindler GmbH, Freiburg, Germany). After deparaffinisation, the slides for EGFR were treated with proteinase K (Code S3020, DakoCytomation, Glostrup, Denmark) for 5 min at room temperature, and those for p-EGFR were treated with EDTA at pH 8 in a microwave oven three times for 5 min at 500 W for antigen retrieval. The primary anti-EGFR antibody (mouse monoclonal, clone H11, code M3563, DakoCytomation) was used at a dilution of 1 : 50 (v/v) and incubated for 1 h at room temperature. The primary anti-phospho-EGFR antibody (rabbit monoclonal, pY1173-EGFR, code no. 4407, Cell Signalling, Danvers, MA, USA) was used at a dilution of 1 : 200 (v/v) and incubated overnight at 4°C. For the detection of antigen-antibody reaction, the UltraVision detection system AP Polymer kit (Cat no. TL-125-AL, Lab Vision, Fremont, CA, USA) was used according to the manufacturer's instructions. Fast red was used as chromogen for 20 min; the sections were counterstained with Mayer's haematoxylin for 3 min, subsequently rinsed in ammonium and finally mounted with glycergel. A positive (an NSCLC tumour specimen with known positivity) and a negative (omission of primary antibody) control were used. The stained sections were independently evaluated by two pathologists (AK and ES). Immunoreactions of EGFR were graded as 3+ when strong complete membrane staining was observed in more than 10% of the tumour cells, as 2+ when more than 10% of the tumour cells showed weak-to-moderate complete membrane staining, as 1+ when partially, faint membrane stain was detected in more than 10% of the tumour cells and as 0 when no staining at all or membrane staining in less than 10% of the tumour cells was observed. In this study, we arbitrarily classified EGFR expression status in two subsets; the 2+ or 3+ signals were considered as EGFR overexpression and the 0 or 1+ signals as non-expression (Koutsopoulos *et al*, 2007).

A paraffin block from the HCC827 lung epithelial adenocarcinoma cell line and commercially available positive controls (SignalSlide Phospho-EGF Receptor (Tyr1173) IHC Controls, code no. 8102, Cell Signalling) was used to validate the antibody for p-EGFR. pY1173-EGFR immunostaining was mainly membranous and was graded as 0 (<5% positive cells), 1+ (5–19% positive cells), 2+ (20–50% positive cells) and 3+ (>50% positive cells) (Cortas *et al*, 2007).

### Statistical analysis

McNemar test was used to compare the *EGFR* and *K-RAS* status between primary tumours and related metastatic sites. Differences were considered statistically significant when the *P*-value was <0.05. All statistical tests were two-sided.

## Results

### Patient characteristics

Twenty-two (88%) patients were men and 22 (88%) were active or former smokers and their median age was 55 years (range, 41–70). Eighteen (72%) patients had adenocarcinomas (ADC) and 21 (84%) patients had stage III or IV disease. Among the 50 samples analysed (25 primary tumours and 25 metastases), 26 (52%) samples were surgical and 24 (48%) biopsies. The primary tumour tissue was the lung (*n*=25 patients); and the origin of the metastatic sample was lung (*n*=9 patients), thoracic wall (*n*=5 patients), adrenal gland (*n*=4 patients), brain (*n*=3 patients), bone (*n*=2 patients), liver (*n*=1 patient) and skin (*n*=1 patient). Metastases were metachronous in all cases; the median time elapsed between resection of the primary tumour and the corresponding metastatic site was 30 months (range, 4–143). The patients' clinicopathologic characteristics are presented in Table 1. Nine (36%) patients were treated with gefitinib in the context of an Expanded Access Program (Table 2).

### *EGFR* mutation status of the primary tumours and the corresponding metastasis

The *EGFR* mutation status of the primary tumours and the corresponding metastases is presented in Table 2. Epidermal growth factor receptor mutations were detected in the primary tumours of five (20%) patients; of these, three of them were the well-characterised ‘hotspot’ mutations in exon 19 (Del746-750 and E746V; case nos. 20, 23 and 18) and the remaining two were novel point mutations in exons 18 and 21 (L692P and G857E; case nos. 17 and 19), respectively. The corresponding metastases of the Del746-750 (case no. 23), E746V (no. 18), L692P (no. 17) and G857E (no. 19) mutant primary tumours were wild type with respect to *EGFR* mutation status. Epidermal growth factor receptor mutations were detected in the metastatic tumours of three (12%) patients (Table 2). The metastasis of one of these patients showed the same *EGFR* mutation (Del19) as the primary tumour as well as an additional one, the T790M in exon 20 (no. 20); conversely, the other two patients carried two novel mutations in exon 18 (L692P and V717A; no. 12) and the T847A (no. 13) in exon 21, which could not be detected in the patients' primary tumour samples. We have confirmed that the non-classical mutations detected in our series are not single nucleotide polymorphisms by mutation analysis of matched normal tissue or blood (data not shown).

Consequently, the *EGFR* gene status could be classified as: (i) *EGFR* wild type in both primary tumour and metastasis (*n*=18 patients; 72%), and (ii) *EGFR* mutations detected only in the primary tumour (*n*=4 patients; 16%) or the metastases (*n*=2 patients; 8%) or both (*n*=1 patient; 4%). Therefore, *EGFR* mutation status showed a discordance of 28% (7 of 25 patients) (McNemar test, *P*=0.688) between the primary tumour and corresponding metastasis (Table 3).

### EGFR and p-EGFR expression by IHC in the primary tumour and the corresponding metastases

Én 19 patients sufficient tumour tissue was available for immunohistochemical analysis of EGFR (Tables 2 and 4). The incidence of EGFR overexpression (grade 2+, 3+) was 26.5% in both primary and metastatic tumours. Concordance between the primary tumour and the corresponding metastases was observed in 17 (89.5) patients (Cohen's Kappa=0.729, *P*=0.001) and among them EGFR was overexpressed in four (21.1%). Discordance was observed in two (10.5%) patients (nos. 14 and 15) (McNemar test, *P*=1). Evaluable paired tissue specimens for p-EGFR analysis by IHC were available in 16 patients. Three out of 16 (18.8%) primary and seven out of 16 (43.8%) metastatic tumours expressed phosphorylated EGFR (pY1173-EGFR-positive). Discordance between the primary and metastatic tumours was observed in eight (50%) patients (McNemar test, *P*=1) (Tables 2 and 4). There was no correlation between the expression of EGFR and pY1173-EGFR. In order to confirm our findings, we repeated the immunohistochemistry from serial sections in all tumour specimens that exhibited EGFR-negative and p-EGFR-positive staining and the obtained results were identical. Epidermal growth factor receptor gene copy number was also investigated by fluorescence *in situ* hybridisation in eight patients (nos. 2, 5, 7, 9, 10, 13, 19 and 24) for whom paired tissue specimens were available; amplification of *EGFR* gene was not detected in any of these tumour specimens.

### *K-RAS* mutation status of the primary tumours and the corresponding metastases

Primary and metastatic tumours were also assessed for *K-RAS* mutations (Table 2). *K-RAS* mutations were detected in the primary tumours of five (20%) patients (nos. 8, 10, 22, 23 and 25) and in the metastatic tumours of five (20%) patients (nos. 9, 10, 11, 16 and 23), respectively. Two patients (nos. 10 and 23) carried the same *K-RAS* mutations in both primary and metastatic tumours (Table 2). One of them (case no. 23) carried the Del746-750 EGFR mutation in the primary tumour but not in metastasis. This was confirmed by three independent PCRs from three genomic DNAs extracted from serial sections of the paraffin blocks. Discordance in *K-RAS* mutation status between the primary tumours and the corresponding metastases was observed in six (24%) patients (McNemar test, *P*=1) (Table 3).

### Response to gefitinib according to *EGFR* and *K-RAS* mutation status

Nine patients received gefitinib as first (nos. 23 and 18), third (nos. 15, 16, 19 20 and 22) or fourth (nos. 14 and 17) line treatment. Three patients received gefitinib before the biopsy of metachronous metastases (nos. 17, 18 and 20) and six received gefitinib after the biopsy of metastases (nos. 14, 15, 16, 19, 22 and 23). Five patients (56%) achieved stable disease and four (44%) progressive disease (Table 2). All patients who experienced progressive disease on gefitinib were wild type regarding the *EGFR* mutation status in both primary tumours and metastases (nos. 14, 15, 16, and 22), of these, two patients carried *K-RAS* mutations in the primary tumour (no. 22) or metastasis (no. 16). All the five patients with stable disease on gefitinib carried EGFR mutations in their primary tumours (nos. 17, 18, 19, 20 and 23). Of these, three patients carried the well-characterised activating mutations in exon 19 (nos. 18, 20 and 23) and two carried *EGFR* mutations of unknown function (nos. 17 and 19). Patient nos. 18 and 20 who received gefitinib before metachronous metastasis developed metastatic tumours, which were either wild type in respect to *EGFR* mutation status (no. 18) or had acquired resistance because of the T790M *EGFR* mutation (no. 20). Patient no. 23, who received gefitinib after metastasis, carried both *EGFR* and *K-RAS* mutations in the primary tumour and the same *K-RAS* mutation in metastasis.

## Discussion

Several studies have shown that activating *EGFR* mutations in exons 18, 19 and 21 have been associated with a 75–95% objective response rate with EGFR TKIs, whereas the *K-RAS* mutations were associated with a lack of sensitivity to these agents (Pao *et al*, 2005; Sharma *et al*, 2007). Epidermal growth factor receptor gene amplification and protein expression have also been considered as predictors of clinical benefit with gefitinib (Hirsch *et al*, 2006). However, in clinical studies, a significant percentage of patients presented clinical benefit when treated with TKIs irrespective of the expression and mutational status of *EGFR* (Sharma *et al*, 2007). As, in most studies, *EGFR* expression and mutations were determined on the primary tumour, the observed clinical benefit of patients with wild-type *EGFR* or the absence of response to TKIs of patients with *EGFR* mutations could be due to discordance in the *EGFR* mutation status or expression between the primary tumour and the corresponding metastasis.

This study demonstrated the existence of a significant discordance of *EGFR* and *K-RAS* mutations occurring in primary tumours and their corresponding metastases in patients with NSCLC. The discordance in *EGFR* mutation status was 28% and that in *K-RAS* was 24%. Similarly, two other studies in paired NSCLC tumours showed a discordance of 32 and 27% regarding the *EGFR* gene copy number (Italiano *et al*, 2006; Bozzetti *et al*, 2008), whereas another study including six NSCLC patients of Asian ethnicity reported a 100% concordance in regard to *EGFR* mutation status (Matsumoto *et al*, 2006). This discrepancy could be related to the different sites of the metastatic tumours analysed in this study (five different distant metastases), whereas in the Matsumoto's *et al* study, only tumour samples from brain metastases were included. Diverse sites of metastases in NSCLC probably represent different clonal outgrowths. Alternatively, we cannot exclude that the different ethnicity of patients and/or the different types of *EGFR* mutations could be the reason for this discrepancy (Tsao *et al*, 2006; Pallis *et al*, 2007). In this study including only Caucasian patients, three classical activating (Del19 and E746V) and four non-classical mutations (T847A, L692P-V717A, L692P and G857E) were detected, whereas in Matsumoto's *et al* study, only classical activating Del19 and L858R mutations were reported. Concerning the non-classical mutations, it is unlikely to represent PCR artifacts, as L692F, T847I and G857E *EGFR* mutations have been previously reported (Fujimoto *et al*, 2005; Tsao *et al*, 2005; Hsieh *et al*, 2006). Furthermore, the expression of phosphorylated EGFR (pY1173-EGFR-positive) on tumour cells from metastatic lesions carrying the T847A and L692P-V717A mutations strongly suggests that the former might be activating EGFR mutation. The time elapsed between diagnoses of the primary tumour and corresponding metastasis in patients (nos. 13 and 12) carrying the abovementioned mutations was 74.5 and 10 months, respectively. Therefore, acquisition of new mutations may be developed during the evolution of the metastatic process.

The administration of TKIs could be an additional explanation of the observed discordance of *EGFR* mutations. Three out of 25 patients received gefitinib before the development of a metachronous metastasis, whereas none of the patients reported in the Matsumoto's *et al* study had exposed to TKIs. It is known that NSCLC patients with EGFR-dependent primary tumours when treated with TKIs can develop metastases, in which either the EGFR signalling is negated or resistance is acquired due to secondary *EGFR* mutations like T790M or MET amplification (Daneshmand *et al*, 2003; Gazdar and Minna, 2005; Engelman *et al*, 2007; Lutterbach *et al*, 2007). It is interesting to note that in patients who had not been exposed to TKIs before biopsy of metastatic lesions, a 18% (4 out of 22 patients) discordance was observed between primary tumours and related metastases. However, a final explanation of the observed discordance, which cannot be excluded, concerns the low-frequency intratumoral heterogeneity for the occurrence of *EGFR* mutations (Sakurada *et al*, 2008).

Our findings concerning the *K-RAS* mutation status are in agreement with a previous study demonstrating that the *K-RAS* mutational status of the primary tumour does not always predict the status of bone metastasis in NSCLC (Badalian *et al*, 2007). A similar phenomenon was also reported in patients with colorectal cancer (Tortola *et al*, 2001). Although *K-RAS* mutations seem to be associated with the early development of NSCLC, it cannot be excluded that *K-RAS* mutations are lost later during tumour progression (Burmer and Loeb, 1989; Li *et al*, 1994). This may, in part, explain the discordance in the *K-RAS* mutation status between primary tumours and metachronous metastases.

Another possibility for the observed discordance in the *EGFR* and *K-RAS* mutation status could be related to the administered chemotherapy. However, as shown in Table 1, among the 10 patients who had not received any treatment before the mutation analysis of the metastatic lesions, 5 developed metastases with different mutation status from that of the corresponding primary tumours. Thus, although tumour clone selection through the various treatments could be an explanation for the different molecular pattern in the primary tumour and metastatic site, our findings suggest that the metastasis genotype could be different from that of the corresponding primary tumour irrespectively of administered chemotherapy.

Previous studies have shown that *EGFR* and *K-RAS* mutations are mutually exclusive, suggesting the presence of different pathways of lung carcinogenesis. However, as previously reported, our data show that *K-RAS* mutation may coexist with *EGFR* mutation (Fujimoto *et al*, 2005; Han *et al*, 2006). Among the five patients with *EGFR* mutations in primary tumours, one patient concomitantly had *K-RAS* mutation (G12C with deletion in exon 19). In the metachronous metastasis, only the *K-RAS* mutation was retained. This patient received gefitinib after the biopsy of metastatic lesion and had stable disease lasting for 3.5 months. The limited duration of response is compatible with the knowledge that the presence of *K-RAS* mutations is associated with resistance to TKIs.

In this study, the expression of pY1173-EGFR was different between primary tumours and corresponding metastases in eight (50%) patients, whereas EGFR expression was discordant in two (10%) patients. The apparent lack of correlation between EGFR and p-EGFR expression has been previously reported and could be due to the different scoring systems and different sensitivities of antibodies used to evaluate the EGFR and p-EGFR expressions (Han *et al*, 2005). It is well known that overexpression of *EGFR* is associated with Tyr phosphorylation of the receptor proteins and that mutations in the kinase domain may cause constitutive phosphorylation of EGFR (Chen *et al*, 2006). However, biochemical studies have shown that variable phosphorylation rates were associated with different tyrosine phosphorylation sites in the receptor; G719S mutant receptor had less EGF-induced phosphorylation at Y845, Y992, Y1068 and Y1173 than did wild-type EGFR, whereas L858R mutant receptor preferentially phosphorylated at the Y1068 but not at Y1173 (Sordella *et al*, 2004; Chen *et al*, 2006; Liu *et al*, 2007).

Clinical investigations suggested a correlation between a high *EGFR* gene copy number and *EGFR* mutations (Cappuzzo *et al*, 2005; Takano *et al*, 2005). Our study failed to demonstrate such a correlation probably due to limited number of cases analysed. Evaluable paired tissue specimens for fluorescence *in situ* hybridisation analysis were available for eight patients. Among them, two (nos 13 and 19) patients presented different *EGFR* mutation status in the primary tumours and corresponding metastases; however, these two patients harbour non-classical *EGFR* mutations (T847A and G857E) and did not show different *EGFR* amplification patterns between paired samples. On the basis of these findings, at the present time, we consider that only the genetic differences unequivocally distinguish EGFR-dependent tumours, which are likely to be sensitive to TKIs from the tumours that could be resistant to these agents (Papadopoulos *et al*, 2006).

In conclusion, our findings indicate a substantial discordance of *EGFR* and *K-RAS* mutations between the primary tumours and the corresponding metastases in NSCLC and underline the need to consider the genotype of both primary and metastatic tumours for selecting patients who will respond to therapy with TKIs.

## Figures and Tables

**Table 1:** Patient's clinicopathological characteristics

**Case**	**Age**	**Sex**	**Histology**	**Stage^a^**	**Smoking status**	**Tissue sample^b^ P^c^/M^d^**	**Metastatic site**	**Time^e^ elapsed between P and M**	**Treatment administered between P and M**
1	60	M	ADC	IV	Active	B/B	Skin	10	Taxane–platinum
2	54	M	SCC	II	Active	S/S	Lung	20	None
3	70	M	ADC	III	Former	B/B	Lung	55	Taxane–gemcitabine
4	44	M	ADC	III	Active	S/B	Lung	65	Taxane–platinum–gemcitabine
5	55	M	ADC	III	Active	S/S	Lung	23	Taxane–platinum
6	63	M	ADC	IV	Active	B/B	Lung	4	None
7	66	M	ADC/BAC	III	Never	S/B	Thoracic wall	12	None
8	57	M	LCC	III	Active	S/B	Thoracic wall	4	Taxane–platinum
9	55	M	ADC	III	Former	S/S	Thoracic wall	15	Taxane–platinum
10	49	M	ADC	II	Active	S/S	Adrenal gland	28	Taxane–platinum
11	50	F	ADC	IV	Active	B/B	Brain	36	Taxane–platinum
12	68	M	ADC	III	Active	S/S	Brain	10	Taxane–platinum
13	44	M	GCC	IV	Active	B/B	Lung	74	Taxane–platinum
14	56	M	ADC	IV	Active	B/S	Adrenal gland	17	None
15	53	M	ADC	III	Active	S/B	Thoracic wall	2	Taxane–platinum
16	41	M	ADC	IV	Active	S/B	Lung	143	None
17	56	M	ADC	IV	Former	B/S	Adrenal gland	36	Taxane–platinum–gemcitabine, gefitinib
18	42	F	ADC	II	Never	S/S	Liver	30	Taxane–platinum, gefitinib
19	55	M	ADC	III	Former	S/B	Bone	2	None
20	46	M	SCC	III	Active	S/B	Lung	45	Taxane–platinum–gefitinib
21	62	M	LCC	III	Never	S/B	Bone	48	Platinum–gemcitabine
22	67	M	ADC	IV	Active	B/S	Adrenal gland	14	None
23	53	M	ADC	IV	Active	S/B	Brain	21	None
24	52	F	ADC/BAC	II	Active	S/S	Lung	51	None
25	63	M	ADC	IV	Active	B/B	Thoracic wall	1	None

ADC=adenocarcinoma; ADC/BAC=adenocarcinoma with bronchoalveolar features; GCC=giant cell carcinoma; LCC=large cell carcinoma; F=female; M=male; SCC=squamous carcinoma.

a Stage, corresponds to that of the time of primary diagnosis.

b Tissue sample: B, biopsy; S, surgery.

c P, primary tumour.

d M, metastasis.

e Time, months.

**Table 2:** *EGFR* and *K-RAS* status in paired primary and metastatic tumours

	***EGFR* mutation status**		**EGFR expression**		**p-EGFR expression**		***K-RAS* mutation status**			
**Case**	**Primary**	**Metastasis**	**Primary**	**Metastasis**	**Primary**	**Metastasis**	**Primary**	**Metastasis**	**Gefitinib**	**Response**
1	wt	wt	1+	1+	0	2+	wt	wt	No	—
2	wt	wt	3+	3+	1+	1+	wt	wt	No	—
3	wt	wt	0	1+	0	0	wt	wt	No	—
4	wt	wt	3+	2+	0	1+	wt	wt	No	—
5	wt	wt	0	0	0	0	wt	wt	No	—
6	wt	wt	1+	0	0	0	wt	wt	No	—
7	wt	wt	0	1+	0	0	wt	wt	No	—
8	wt	wt	1+	0	0	0	G12S	wt	No	—
9	wt	wt	1+	1+	0	1+	wt	G13S	No	—
10	wt	wt	2+	3+	0	0	G12V	G12V	No	—
11	wt	wt	ND	2+	ND	2+	wt	G12S	No	—
12	wt	L692P V717A	1+	0	ND	2+	wt	wt	No	—
13	wt	T847A	0	0	0	1+	wt	wt	No	—
14	wt	wt	3+	0	1+	0	wt	wt	Yes	PD
15	wt	wt	0	2+	ND	2+	wt	wt	Yes	PD
16	wt	wt	0	0	0	2+	wt	G12A	Yes	PD
17	L692P	wt	0	1+	0	0	wt	wt	Yes	SD
18	E746V	wt	0	0	ND	0	wt	wt	Yes	SD
19	G857E	wt	0	0	0	1+	wt	wt	Yes	SD
20	E746-A750 del	E746-A750 del T790M	2+	3+	2+	0	wt	wt	Yes	SD
21	wt	wt	ND	ND	ND	ND	wt	wt	No	—
22	wt	wt	ND	ND	ND	ND	G12D	wt	Yes	PD
23	E746-A750 del	wt	ND	ND	ND	ND	G12C	G12C	Yes	SD
24	wt	wt	ND	ND	ND	ND	wt	wt	No	—
25	wt	wt	ND	ND	ND	ND	G12C	wt	No	—

**Table 3:** Combined analysis of *EGFR* and *K-RAS* mutation status

	**Primary/metastatic tumour**				
	**wt/wt**	**wt/mut**	**mut/wt**	**mut/mut**	**Discordance**
EGFR	18 (72%)	2 (8%)	4 (16%)	1 (4%)^a^	7 cases (28%)
K-RAS	17 (68%)	3 (12%)	3 (12%)	2 (8%)	6 cases (24%)

a Del19/Del19 and T790M.

**Table 4:** EGFR expression as assessed by IHC

	**Primary tumour status/metastasis status**				
	**+ve/+ve**	**−ve/−ve**	**+ve/−ve**	**−ve/+ve**	**Discordance**
EGFR	4 (21.1%)	13 (68.4%)	1 (5.3%)	1 (5.3%)	2/19 (10.6%)
p-EGFR (Y1173)	1 (6.2%)	7 (43.8%)	2 (12.5%)	6 (37.5%)	8/16 (50%)

IHC=immunohistochemistry.
